# Supplementary material for: “In Initiative Overload”: Australian Perspectives on Promoting Physical Activity in the Workplace from Diverse Industries
Source: Int J Environ Res Public Health. 2019 Feb 12;16(3):516. doi: 10.3390/ijerph16030516 (PMC6388384; doi:10.3390/ijerph16030516)
Supplement: Supplementary file 1 [file ijerph-16-00516-s001.pdf]

Supplementary Materials S1. Employee focus group and manager interview question guides

The following questions (and clarification prompts) were used to guide the focus group discussions:

- What does a typical day look like in terms of movement at this workplace? (Do you spend most of your time sitting, standing, moving about, doing tiring work e.g. lifting, carrying?)
- Do you think there might be any problem with this pattern (e.g. Health risks? What do you understand about these? Too much sitting/standing in one position? Breaking sitting time? Changing posture? Is too much sitting/standing in one place bad for health? How? Why? etc)
- Do you think anything should and can be done to change your sitting/standing/moving patterns at work? Is this issue 'off the radar'?
- What would be the main barriers to making change?
- What could help with making changes?
- How would you describe the overall culture in relation to workplace health and safety and wellness?
- Do you consider the issue of too much sitting / standing during your non-work hours? (Do you use active transport to get to work, or compensate for sitting or active work when you are at home?)

The following guide questions were used in the manager interviews:

- How would you describe your workplace environment and culture as it relates to health and wellness?
- Is physical activity/lack of activity in this workplace an issue for employee health? (If yes, what is the nature of the problem?)
- Whose responsibility is it (employer/employee) to ensure optimal movement patterns at work?
- Is anything currently being done to promote more (healthy) movement at this workplace?
- What else could be done to improve the situation in this workplace? What would help to get change?
- What are the main barriers to change?
